# *Ex Vivo* Generation of Human Alloantigen-Specific Regulatory T Cells from CD4^pos^CD25^high^ T Cells for Immunotherapy

**DOI:** 10.1371/journal.pone.0002233

**Published:** 2008-05-21

**Authors:** Jorieke H. Peters, Luuk B. Hilbrands, Hans J. P. M. Koenen, Irma Joosten

**Affiliations:** 1 Department of Bloodtransfusion and Transplantation Immunology, Radboud University Nijmegen Medical Centre, Nijmegen, The Netherlands; 2 Department of Nephrology, Radboud University Nijmegen Medical Centre, Nijmegen, The Netherlands; New York University School of Medicine, United States of America

## Abstract

**Background:**

Regulatory T cell (Treg) based immunotherapy is a potential treatment for several immune disorders. By now, this approach proved successful in preclinical animal transplantation and auto-immunity models. In these models the success of Treg based immunotherapy crucially depends on the antigen-specificity of the infused Treg population. For the human setting, information is lacking on how to generate Treg with direct antigen-specificity *ex vivo* to be used for immunotherapy.

**Methodology/Principal Findings:**

Here, we demonstrate that in as little as two stimulation cycles with HLA mismatched allogeneic stimulator cells and T cell growth factors a very high degree of alloantigen-specificity was reached in magnetic bead isolated human CD4^pos^CD25^high^ Treg. Efficient increases in cell numbers were obtained. Primary allogeneic stimulation appeared a prerequisite in the generation of alloantigen-specific Treg, while secondary allogeneic or polyclonal stimulation with anti-CD3 plus anti-CD28 monoclonal antibodies enriched alloantigen-specificity and cell yield to a similar extent.

**Conclusions/Significance:**

The *ex vivo* expansion protocol that we describe will very likely increase the success of clinical Treg-based immunotherapy, and will help to induce tolerance to selected antigens, while minimizing general immune suppression. This approach is of particular interest for recipients of HLA mismatched transplants.

## Introduction

Regulatory T cells (Treg) play a critical role in various immunological processes. These cells dampen immune responses, which is important in maintenance of (self-) tolerance and homeostasis. Immunotherapy based on Treg (either in vivo facilitation of Treg or infusion of *ex vivo* isolated / manipulated Treg) is therefore a potential treatment for many immune disorders. Such an approach has been proven successful in animal models of stem cell transplantation [1]–[7], solid organ transplantation [8], [9], auto-immunity [10]–[14] and even in infertility [15]. Treg therapy in human patients will require high cell numbers, which might be obtained by *ex vivo* expansion. It has been shown that human naturally occurring CD4^pos^CD25^high^ Treg can be expanded polyclonally, by using anti-CD3 and anti-CD28 antibody stimulation in combination with IL-2 and/or IL-15 [16]–[21]. These polyclonal expansion protocols greatly increase Treg numbers, while preserving suppressive capacity. However, there are some drawbacks regarding clinical application of polyclonally expanded Treg. First, since this type of activation encompasses Treg with a broad range of specificities, the infused polyclonal Treg may suppress immune responses other than the target response, thereby increasing the risk for opportunistic infections and tumor-growth. Second, due to the low percentage of Treg within a polyclonal cell pool that is specific for a given target antigen, large numbers of Treg need to be infused. These difficulties could be overcome by using antigen-specific Treg. In animal models, antigen-specific Treg have been shown to be far more efficient than polyclonal Treg [4], [6], [7], [12]–[14]. Even as few as 5×10^3^ antigen-specific Treg were sufficient to prevent the onset of diabetes in a NOD model [14].

For the human setting, information is scarce on how to generate sufficient numbers of antigen-specific Treg ex-vivo from naturally occurring Treg [22]. The aim of this study was to design a successful strategy to obtain high numbers of Treg with direct alloantigen-specificity for use in transplantation settings. Based on previous findings, we hypothesized that it would be beneficial to combine polyclonal stimulation, to boost expansion, with alloantigen stimulation, to increase antigen-specificity. To test this hypothesis, we alternated these two stimulation methods in primary and secondary Treg expansion cycles and assessed cell numbers, phenotype, function and antigen-specificity before and after expansion. Thus, we succeeded in defining a protocol that yields high numbers of strictly alloantigen-specific human Treg.

## Results

### Optimal conditions for primary and secondary expansion cycles

**Figure 1:**
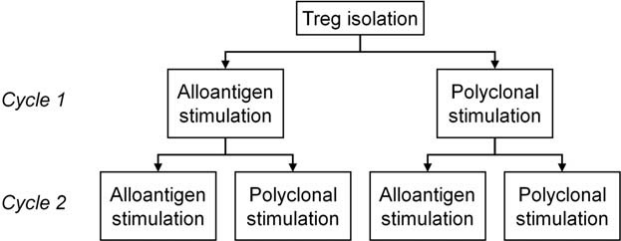
Schematic overview of expansion strategies. Treg were expanded in two cycles, in which alloantigen and polyclonal stimulation was alternated, resulting in four distinct strategies: two subsequent cycles with alloantigen stimulation; primary cycle with alloantigen stimulus and secondary cycle with polyclonal stimulus; primary cycle with polyclonal stimulus and secondary cycle with alloantigen stimulus; and two subsequent cycles with polyclonal stimulation.

With the objective to obtain the highest numbers of functionally active Treg with optimal direct alloantigen-specificity, we devised four expansion strategies, employing polyclonal and alloantigen-specific stimulation in two subsequent cycles of Treg expansion (Figure 1). The cells that were obtained with these expansion strategies were compared in terms of absolute cell numbers, phenotype, suppressive capacity, antigen-specificity and anergy. Prior to analysis of the four selected Treg expansion strategies, individual expansion cycles were optimized, with regard to strength and mode of stimulation.

**Figure 2:**
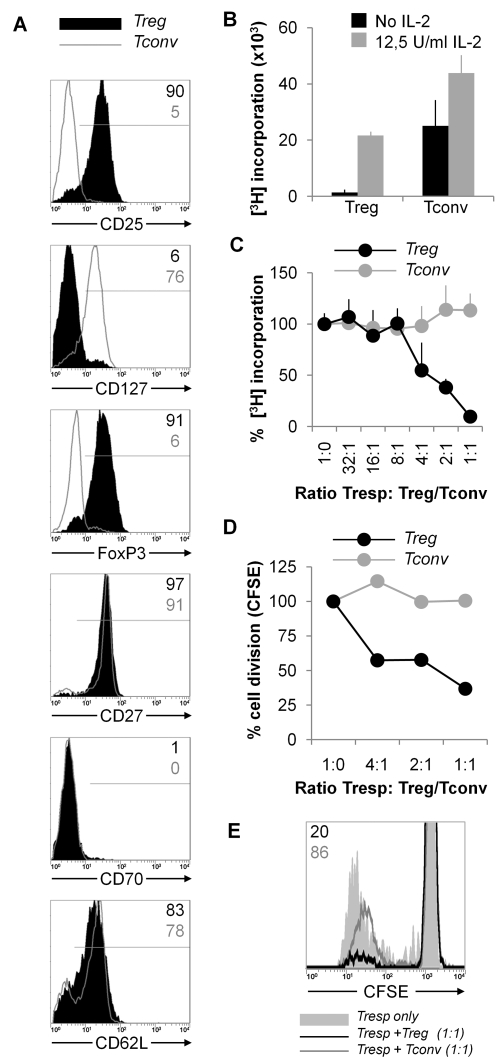
Phenotypical and functional characteristics of freshly MACS-isolated Treg. CD4^pos^ T cells were negatively isolated from PBMC and separated into CD25^pos^ (Treg) and CD25^neg^ (Tconv) T cell fractions by magnetic cell sorting. Data from a typical isolation is shown. (A) Cell surface expression of CD25, CD127, CD27, CD70 and CD62L, and intracellular expression of FoxP3 were analyzed on Treg (black filled histograms) or Tconv (grey line histogram). (B) Proliferative capacity of Treg or Tconv upon stimulation with HLA mismatched gamma irradiated allogeneic PBMC was determined in the absence (black bar) or presence (grey bar) of exogenous IL-2. Proliferation was determined by measuring [^3^H]Thymidine incorporation at day 5. (C) Suppressive capacity of Treg in alloantigen responses was determined in a MLR co-culture using [^3^H]Thymidine incorporation. Autologous naïve CD4^pos^CD25^neg^ Tresp cells were stimulated with HLA mismatched gamma irradiated allogeneic PBMC. Treg (black lines) or Tconv (grey lines) were added into these cultures at indicated Tresp∶Treg/Tconv ratios. Proliferation was determined by measuring [^3^H]Thymidine incorporation at day 5. Results are expressed as percentage of [^3^H]Thymidine incorporation +SD, indexed to [^3^H]Thymidine incorporation of naïve Tresp and antigen only. (D) Suppressive capacity of Treg in alloantigen responses was determined in a MLR co-culture using CFSE dilution. CFSE labeled autologous naïve CD4^pos^CD25^neg^ Tresp cells were stimulated with PKH26 labeled HLA mismatched gamma irradiated allogeneic PBMC. Treg (black lines) or Tconv (grey lines) were added into these cultures at indicated Tresp∶Treg/Tconv ratios. Proliferation was determined by measuring CFSE dilution of Tresp at day 5. Results are expressed as percentage of proliferating cells, indexed to percentages of proliferating cells in cultures of naïve Tresp and antigen only. (E) Example CFSE of suppression assay as described in Figure 2D. Tresp only (grey filled histogram), Tresp+Treg (1∶1, black line) and Tresp+Tconv (1∶1, grey line) are shown, numbers indicate percentage of proliferating cells, indexed to percentages of proliferating cells in cultures of naïve Tresp and antigen only.

Cell sorting based on multiple surface markers such as CD25, CD127, CD62L and CD27 yields a high purity of FoxP3^pos^ Treg. However, these tools are not available for clinical grade purposes. Therefore, we specifically chose to use magnetic bead isolation for purification of Treg, as this most closely fits with the currently available GMP isolation tools. Cells isolated by this procedure expressed CD25 and FoxP3, but not CD127 (Figure 2A), thus displaying a typical Treg phenotype [23]–[26]. In contrast, CD4^pos^CD25^neg^ conventional T cells (Tconv) did not express FoxP3 or CD25, and were positive for CD127. The majority of the isolated Treg as well as Tconv expressed the differentiation marker CD27, not its ligand CD70, and were positive for CD62L.

The freshly isolated Treg were hyporesponsive upon restimulation (Figure 2B) and able to suppress proliferation of autologous CD4^pos^CD25^neg^ T cells upon stimulation with alloantigen (Figure 2C, D and E), >50% suppression was seen with Tresp∶Treg ratios of 4∶1 or lower (Figure 2C). Tconv were not anergic, nor suppressive.

**Figure 3:**
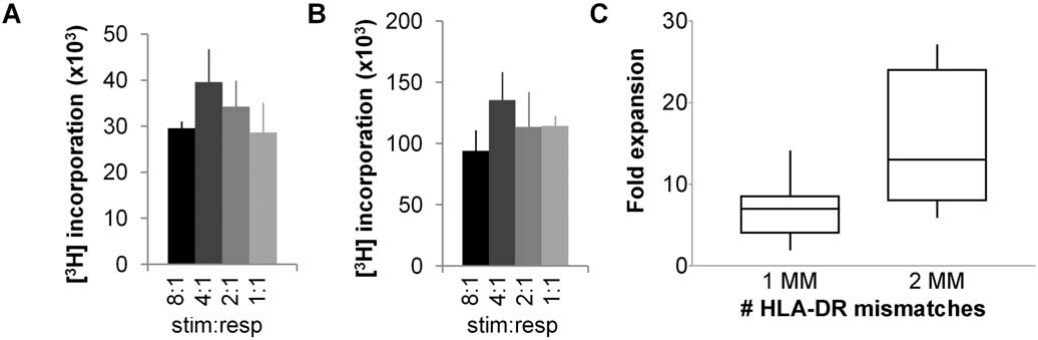
Determination of optimal strength of alloantigen Treg stimulation. (A) Treg were stimulated with indicated ratios of irradiated allogeneic PBMC in the presence of IL-2 and IL-15. Proliferation was determined by measurement of [^3^H]Thymidine incorporation at day 5. Data are representative of three independent experiments. (B) Treg primed with alloantigen were restimulated with indicated ratios of irradiated allogeneic PBMC in the presence of IL-2 and IL-15. Proliferation was determined by measurement of [^3^H]Thymidine incorporation at day 3. Data are representative of three independent experiments. (C) Efficiency of primary alloantigen stimulated expansion is related to the number of HLA-DRB1 mismatches. Freshly isolated Treg were expanded in one cycle in the presence of IL-2 and IL-15 and stimulation by gamma irradiated allogeneic PBMC with one (N = 10) or two (N = 6) mismatches on HLA class II DRB1 genes. Expansion values were calculated by relating the number of cells set up in the initial culture to the number of cells after expansion (after two days rest). Expansion was higher with alloantigen mismatched on two HLA-DRB1 genes as compared to alloantigen with one mismatch. This difference was found to be statistically significant in a Mann Whitney test (P = 0.025).

For alloantigen driven expansion, the optimal strength of alloantigen stimulation was determined by titrating irradiated HLA mismatched allogeneic PBMC into Treg cultures, in the presence of exogenous IL-2 and IL-15. In a primary MLR, proliferation was maximal with stimulator∶responder ratios of 4∶1 (Figure 3A). Secondary stimulation with alloantigen showed similar results (Figure 3B). The data shown in Figure 3B were obtained with Treg primed with alloantigen, but similar results were observed when polyclonally primed Treg were used (data not shown). Consequently, for expansion experiments, a stimulator∶responder ratio of 4∶1 was used. Interestingly, a higher number of HLA-DRB1 mismatches between stimulator and responder resulted in higher expansion rates (P<0.05) (Figure 3C). Mismatches at other HLA genes did not significantly correlate with expansion efficiency (data not shown).

**Figure 4:**
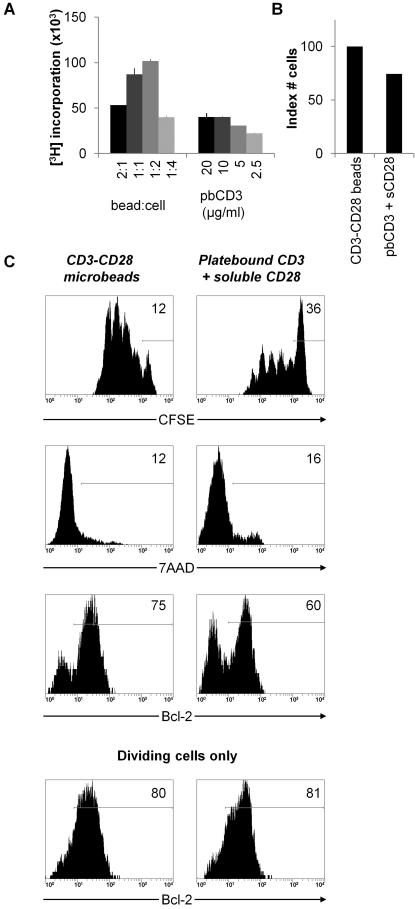
Determination of optimal mode and strength of primary polyclonal Treg stimulation. (A) Treg were stimulated with indicated ratios of anti-CD3+ anti-CD28 microbeads or with indicated concentrations of platebound anti-CD3+ soluble anti-CD28 (1 μg/ml) in the presence of IL-2 and IL-15. Proliferation was determined by measurement of [^3^H]Thymidine incorporation at day 4. (B) Indexed cell yield after primary polyclonal Treg culture using optimal strength of anti-CD3+ anti-CD28 microbeads (set to 100%) or platebound anti-CD3 plus soluble anti-CD28 stimulation. (C) Cell division pattern and survival/death signals of Treg after different primary polyclonal stimulations. Treg were stained with CFSE and stimulated with optimal strength of anti-CD3+ anti-CD28 microbeads or platebound anti-CD3+ soluble anti-CD28. CFSE dilution pattern, 7AAD staining and Bcl-2 expression of Treg population were assessed at day 4. In the lowest row, Bcl-2 expression is depicted gated on dividing Treg only. Data are representative of four independent experiments.

For polyclonal stimulation we used CD3 and CD28 triggering, and we compared anti-CD3+ anti-CD28 mAb coated microbeads with platebound anti-CD3+ soluble anti-CD28 mAb stimulation. For primary expansion of Treg, higher proliferation rates were achieved with beads as compared to platebound anti-CD3+ soluble anti-CD28 mAb (Figure 4A). Cell yield after expansion of Treg with either stimulation mode, using optimal dosage, was higher for anti-CD3+ anti-CD28 bead stimulation as compared to platebound anti-CD3+ soluble anti-CD28 stimulation (Figure 4B). To explain these differences, we assessed cell division and survival in Treg cultures for both stimuli (Figure 4C). Upon stimulation with anti-CD3+ anti-CD28 mAb coated beads, the majority of cells were triggered to proliferate as determined by CFSE dilution. Expression of 7AAD, a marker for late apoptotic cells, was low. Bcl-2, an anti-apoptotic protein, was expressed by 70–85% of the cells. Further gating revealed that the dividing cell population specifically expressed Bcl-2. Upon stimulation with platebound anti-CD3+ soluble anti-CD28 mAb, and independent of the concentration used, a large portion of cells was not triggered to proliferate. Again 7AAD staining was low. The percentage of cells that expressed Bcl-2 was lower as compared to cells stimulated with anti-CD3+ anti-CD28 beads, this correlated with the percentage of dividing cells. The results indicate that the lower cell yield after stimulation of Treg with platebound anti-CD3+ soluble anti-CD28 mAb is merely caused by less efficient triggering of Treg proliferation, and is not the result of enhanced cell death.

**Figure 5:**
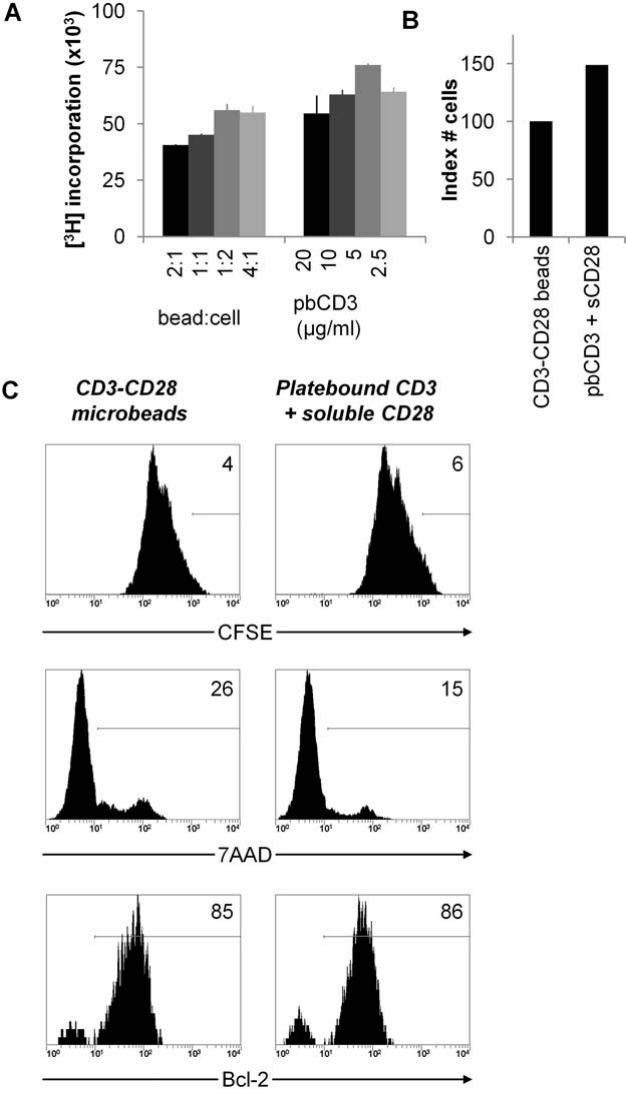
Determination of optimal mode and strength of secondary polyclonal Treg stimulation. (A) Treg were primed with anti-CD3+ anti-CD28 microbeads and restimulated with indicated ratios of anti-CD3+ anti-CD28 microbeads or with indicated concentrations of platebound anti-CD3+ soluble anti-CD28 in the presence of IL-2 and IL-15. Proliferation was determined by measurement of [^3^H]Thymidine incorporation at day 3. (B) Indexed cell yield after secondary polyclonal Treg culture using optimal strength of anti-CD3+ anti-CD28 microbeads (set to 100%) or platebound anti-CD3+ soluble anti-CD28 stimulation. (C) Cell division pattern and survival/death signals of Treg after different secondary polyclonal stimulations. Treg were stained with CFSE and stimulated with optimal strength of anti-CD3+ anti-CD28 microbeads or platebound anti-CD3+ soluble anti-CD28. CFSE dilution pattern, 7AAD staining and Bcl-2 expression of Treg population were assessed at day 4. Data are representative of four independent experiments.

These experiments were also conducted for secondary polyclonal stimulation. In this situation, platebound anti-CD3+ soluble anti-CD28 stimulation was superior as a secondary stimulus as compared to anti-CD3+ anti-CD28 microbead stimulation (Figure 5A). Expansion cultures stimulated with platebound anti-CD3+ soluble anti-CD28 yielded more Treg as compared to cultures stimulated with anti-CD3+ anti-CD28 beads (Figure 5B). Attempting to explain these findings, we again assessed cell division and survival in Treg cultures using both stimuli (Figure 5C). In contrast to the results after primary stimulation, both polyclonal stimuli now induced massive proliferation as assessed by CFSE dilution. Also, both 7AAD staining and Bcl-2 expression were comparable between Treg cultures stimulated with either anti-CD3+ anti-CD28 beads or platebound anti-CD3+ soluble anti-CD28 mAb. The data shown in Figure 5 were obtained with Treg primed with anti-CD3+ anti-CD28 beads, similar results were observed when alloantigen primed Treg were used (data not shown). Overall, these data prompted us to use platebound CD3+ soluble CD28 mAb for polyclonal Treg restimulation in further experiments.

Several reports have described the use of high dose IL-2 in Treg expansion protocols [16], [17]. Our laboratory and others have shown that combination of IL-2 and IL-15 increases Treg proliferation [21], [27]. We thus added titrated concentrations of IL-2 (range 12,5 U/ml to 200 U/ml) with a fixed concentration of IL-15 (10 ng/ml) to Treg cultures to determine the optimal IL-2 dose in our system. Concentrations of 25 U/ml or higher gave similar results in terms of Treg yield (data not shown). We therefore chose to use 25 U/ml IL-2 in combination with 10 ng/ml IL-15 for expansion experiments.

**Figure 6:**
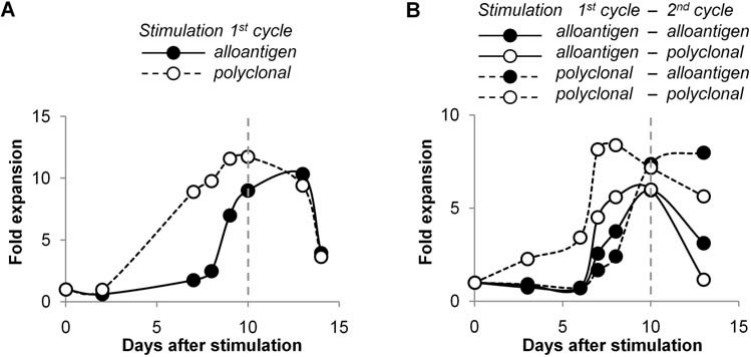
Determination of optimal length of primary and secondary Treg expansion cycles. (A) Optimal length of primary Treg expansion cycles. Treg were stimulated with CFSE-labeled alloantigen (solid line, solid circle) or polyclonal (dotted line, open circle) stimulus in the presence of IL-2 and IL-15. At indicated time points, Treg numbers were counted by FACS (excluding CFSE^pos^ allogeneic stimulator cells) and related to Treg numbers at initial set up. (B) Optimal length of secondary Treg expansion cycles. Treg were primed with alloantigen (solid lines) or polyclonal (dotted lines) stimulation as indicated in the presence of IL-2 and IL-15 and rested for 2 days prior to restimulation. Cells were restimulated with CFSE-labeled alloantigen (solid circles) or polyclonal stimulus (open circles) in the presence of IL-2 and IL-15. At indicated time points, Treg numbers were counted by FACS (excluding CFSE^pos^ allogeneic stimulator cells) and related to Treg numbers at initial set up. Data are representative of three independent experiments.

To determine the optimal cycle length, cell numbers were counted at several time points during primary and secondary expansion cycles. Expansion values were calculated and are depicted in Figure 6. In primary Treg expansion cycles with either alloantigen or polyclonal stimulation, cultures reached maximal cell numbers after 10–12 days. In secondary expansion cycles with alloantigen stimulation, peak cell numbers were found at day 10. In secondary expansion cycles with polyclonal stimulation, peak cell numbers were found at day 10 if cells had been primed with alloantigen. However, when Treg had been primed with polyclonal stimulation, a plateau in cell numbers in a secondary polyclonal stimulation cycle was reached around day 7. Since our prime interest was to obtain alloantigen-specific Treg, the strategy comprising two subsequent cycles with polyclonal stimulation actually served as a control. Consequently, we standardized the expansion cycle length at 10 days for all strategies.

### Cell yield after Treg expansion with combined alloantigen and polyclonal stimulation strategies

**Figure 7:**
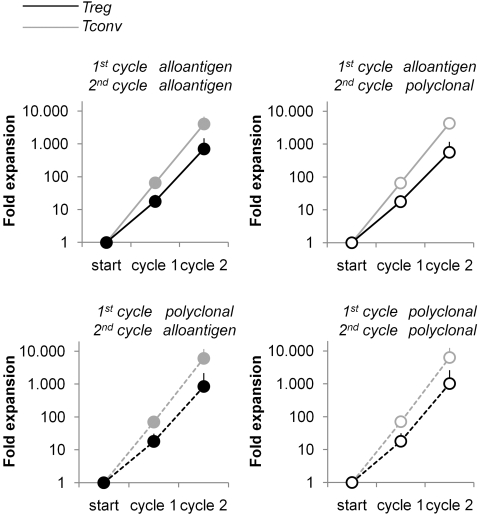
Treg expansion after primary and secondary cycles with alternated alloantigen or polyclonal stimulation. Treg and Tconv were expanded according the schedule in Figure 1 and rested for two days. Treg numbers were determined and related to Treg numbers at initial set up. Data represent average expansion +SD of seven independent experiments.

Having optimized the conditions for the primary and secondary expansion cycles, we now isolated Treg and Tconv and subsequently applied multiple expansion cycles according to the strategies depicted in Figure 1. Average Treg expansion values are shown in Figure 7. Contrary to our expectation that stimulation cycles with alloantigen would provide lower cell yields as compared to cycles of polyclonal expansion, Treg numbers obtained after each of the four expansion strategies were similar (average fold expansion 780, SD 300, p = 0.71, N = 4).

### Phenotypic characterization of expanded Treg

**Figure 8:**
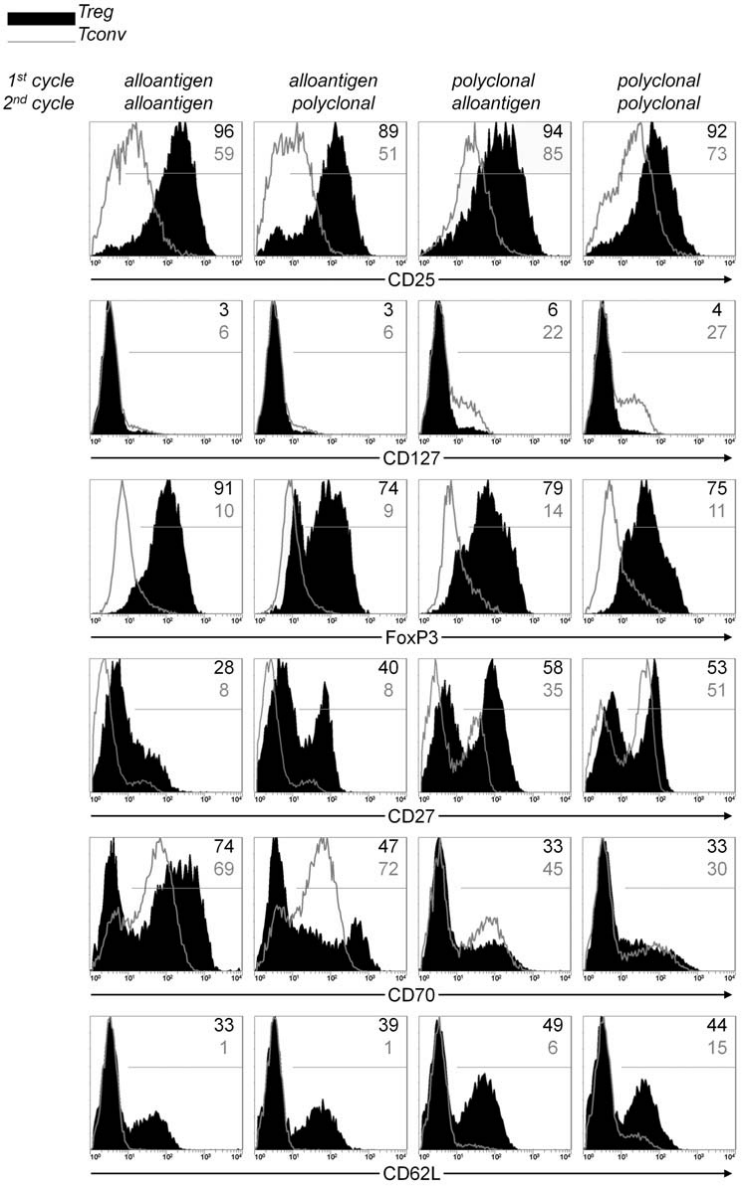
Phenotypical characterization of expanded Treg. Treg and Tconv were expanded according the schedule in Figure 1 and rested for two days. Cell surface expression of CD25, CD127, CD27, CD70 and CD62L, and intracellular expression of FoxP3 was analyzed on Treg (black filled histograms) or Tconv (grey line histogram). Data are representative of four to seven independent experiments.

A potential risk of Treg expansion is the outgrowth of contaminating cell types such as CD8^pos^ T cells or NK-cells. In our experiments, we did not find major contaminations, since typically >90% of the expanded cells were CD4^pos^ T cells. The majority of Treg expanded with either strategy was CD25^high^, and CD127^neg^ (Figure 8). The majority of Treg retained expression of FoxP3^pos^ after two cycles of expansion. Tconv cells were largely FoxP3^neg^. Previously, we and others have shown that Treg constitutively expressing CD27 are stronger suppressors [17], [27]. Typically, after expansion, a portion of Treg retained a CD27^pos^ phenotype, while the remaining Treg shifted towards a CD27^neg^ phenotype. Treg expanded with two cycles of polyclonal stimulation repeatedly showed the highest percentage of CD27^pos^ cells (50 to 80%), while cells expanded with two subsequent alloantigen stimulation cycles had the lowest percentage of CD27^pos^ cells (10 to 40%). The two strategies with alternated alloantigen and polyclonal stimulation cycles showed intermediate percentages of CD27^pos^ cells. The expression of CD70, the ligand for CD27, was reversely correlated with CD27 expression. CD62L is also described as a marker for Treg with high suppressive potency [24]. Whereas expression of CD62L was lost on expanded Tconv cells, a substantial portion of Treg retained expression of this marker.

### Functional characterization of expanded Treg

#### Treg retain anergic properties after expansion

**Figure 9:**
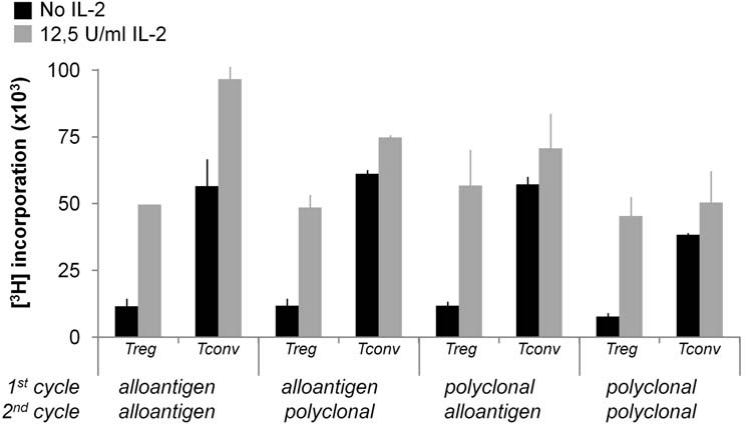
Proliferative capacity of expanded Treg. Treg and Tconv were expanded according the schedule in Figure 1 and rested for two days. Proliferative capacity of expanded cells upon restimulation with target alloantigen (same as used for expansion) was determined in the absence (black bars) or presence (grey bars) of IL-2. Proliferation was determined by measuring [^3^H]Thymidine incorporation at day 3. Results are expressed as percentage of [^3^H]Thymidine incorporation +SD. Data are representative of four independent experiments.

One of the hallmarks of Treg is their anergic (hyporesponsive) behavior *in vitro*. Treg retained their anergic state after expansion, irrespective of the strategy employed, while in contrast Tconv were not anergic. Indeed, Treg did not proliferate upon stimulation in absence of exogenous T cell growth factors, while addition of IL-2 restored the proliferative capacity (Figure 9).

#### Expanded Treg show enhanced suppressive capacity and can acquire full antigen-specificity within two expansion cycles

The main characteristics of alloantigen-specific Treg should be their strong suppressive capacity when stimulated by target antigen (in transplantation settings this would be donor antigen), in parallel with a lack of suppressive activity in the case of stimulation by other antigens such as fully HLA mismatched third party alloantigen. To assess suppressive capacity, we used both the classic [^3^H]Thymidine incorporation co-culture suppression assay as well as a CFSE based assay. The latter is used because in some instances it is difficult to draw clear conclusions from the [^3^H]Thymidine assay when using multiple cell populations.

**Figure 10:**
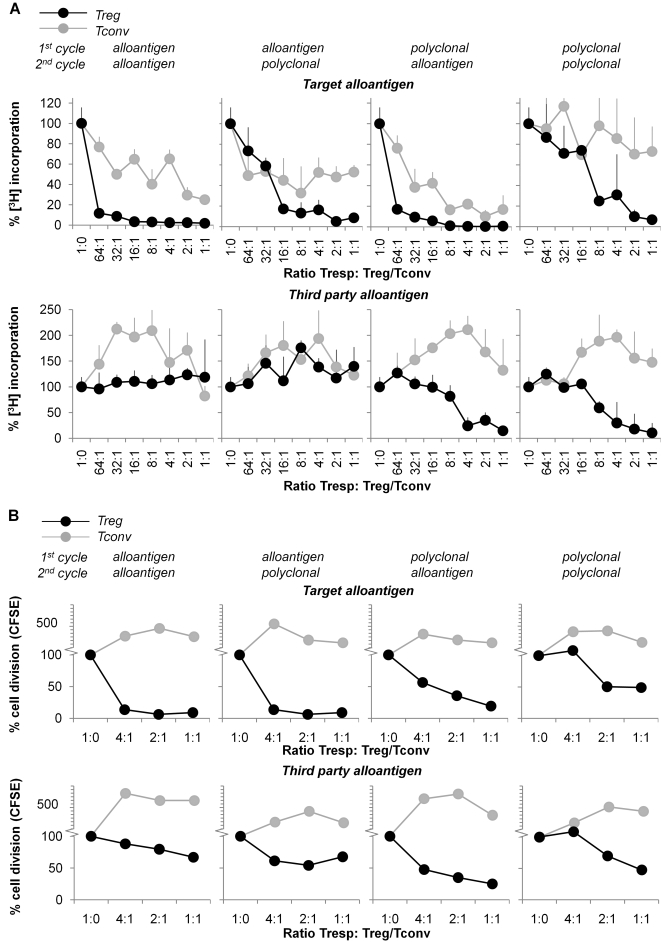
Suppressive capacity of expanded Treg in [^3^H]Thymidine incorporation suppression assays. Treg and Tconv were expanded according the schedule in Figure 1 and rested for two days. (A) Suppressive capacity of Treg in target alloantigen and third party alloantigen responses as determined in a MLR co-culture using [^3^H]Thymidine incorporation. Autologous naïve CD4^pos^CD25^neg^ Tresp cells were stimulated with target alloantigen (same as used for expansion) or third party HLA mismatched gamma irradiated allogeneic PBMC. Expanded Treg (black lines) or Tconv (grey lines) were added into these cultures at indicated Tresp∶Treg/Tconv ratios. Proliferation was determined by measuring [^3^H]Thymidine incorporation at day 5. Results are expressed as percentage of [^3^H]Thymidine incorporation +SD, indexed to [^3^H]Thymidine incorporation of naïve Tresp and antigen only. Data are representative of seven independent experiments. (B) Suppressive capacity of Treg in target alloantigen and third party alloantigen responses as determined in a MLR co-culture using CFSE dilution. CFSE labeled Autologous naïve CD4^pos^CD25^neg^ Tresp cells were stimulated with PKH26 labeled target alloantigen (same as used for expansion) or PKH26 labeled third party HLA mismatched gamma irradiated allogeneic PBMC. Expanded Treg (black lines) or Tconv (grey lines) were added into these cultures at indicated Tresp∶Treg/Tconv ratios. Proliferation was determined by measuring CFSE dilution of Tresp at day 5. Results are expressed as percentage of proliferating cells, indexed to percentages of proliferating cells in cultures of naïve Tresp and antigen only. Data are representative of three independent experiments.

Figure 10 shows that highly antigen-specific Treg were obtained in two situations. Treg expanded with two cycles of alloantigen stimulation showed very high suppressive activity in target antigen driven MLR (>90% suppression in all ratios tested, Figure 10A) and hardly any suppression of third party antigen driven MLR. The cells obtained after the strategy starting off with a primary allostimulation, followed by secondary polyclonal stimulation, also yielded potent Treg with high antigen-specificity, as indicated by strong suppression of target antigen driven responses (>50% suppression with Tresp∶Treg ratios of 16∶1 or lower, Figure 10A) and low inhibition of third party HLA mismatched allostimulated MLR. Importantly, these data show that under these circumstances a very high degree of antigen-specificity can already be obtained after two cycles of expansion. This is relevant since we have noticed that multiple expansion cycles, irrespective of the mode of stimulation, can lead to reduced suppressive capacity, cell exhaustion, and subsequent cell death (data not shown). The expansion strategy starting off with a primary polyclonal stimulus followed by a secondary alloantigen stimulus yielded very potent suppressors, showing >90% suppression of target antigen driven MLC at all ratios tested (highest ratio tested 32∶1, Figure 10A). However, this cell population was still capable of suppression in third party alloantigen driven responses, albeit at a much lower efficiency than target antigen driven responses (>50% suppression at Tresp∶Treg ratios 2∶1 or lower, Figure 10A), indicating a strong enrichment Treg specific for target antigen, but incomplete exclusion of Treg with other specificities. As expected, Treg expanded with two cycles of polyclonal stimulation showed suppression of both target antigen as well as 3^rd^ party alloantigen driven MLR to a similar, moderate degree (>50% suppression at 8∶1 and 4∶1 or lower, respectively, Figure 10A). Treg displayed similar suppressive activity in CFSE suppression assays, while expanded Tconv were not suppressive (Figure 10B). Note that in the [^3^H]Thymidine based suppression assays (Figure 10A), the low counts observed after titration of high numbers of expanded Tconv are not indicative of suppression per se, but rather a result of the very early proliferation of already primed Tconv cells in the co-culture. These cells heavily compete for medium components with the naïve Tresp cells that respond later in time. Tconv present in substantial numbers may indeed lead to distinct culture kinetics with an earlier proliferation peak and a net result of lower counts at day 5.

In summary, optimal results were obtained with Treg expansion in two subsequent cycles of alloantigen stimulation: efficient increases in cell numbers, yielding highly potent and strictly antigen-specific Treg. The success of Treg expansion with alternated alloantigen and polyclonal stimulation depends on the order of the stimulation cycles. When Treg are expanded by a primary alloantigen stimulus followed by a secondary polyclonal stimulus, cells expand efficiently and show highly potent, strictly antigen-specific suppressive capacity. The reverse strategy with a primary polyclonal stimulus and a secondary alloantigen stimulus, yield high numbers of cells, and enrichment for target antigen-specific Treg. However, Treg specific for other antigens are still present. As expected, two cycles of polyclonal stimulation yielded high numbers of Treg. These cells were suppressive, but no enrichment for alloantigen-specific Treg had taken place.

## Discussion

High numbers of Treg will be needed for effective Treg immunotherapy in humans to facilitate tolerance in patients with auto-immunity or after transplantation. *Ex vivo* Treg expansion can provide the solution to obtain these high numbers. Therapeutic efficiency can be improved by selecting for target-antigen reactive Treg, as indicated by preclinical mouse models designed to study the prevention of autoimmunity [12]–[14] and graft-versus-host disease [4], [6], [7]. At the same time, exclusion of Treg with specificities for other antigens lowers the risk for unwanted non-specific immune suppression, thus decreasing the risk on opportunistic infections and tumor growth.

Information on strategies for large scale antigen-specific expansion of human Treg is scant [22], as previous studies on *ex vivo* Treg expansion have primarily focused on polyclonal stimulation of Treg [16]–[21]. In the current report, we demonstrate the *ex vivo* generation of Treg with direct alloantigen-specificity from human naturally occurring CD4^pos^CD25^high^ Treg in as little as two stimulation cycles, requiring primary stimulation with HLA-mismatched allogeneic stimulator cells and IL-2 plus IL-15. Experiments were performed with alternating allogeneic and polyclonal (anti-CD3 anti-CD28 mAb) stimulation to define the *ex vivo* conditions resulting in optimal enrichment of alloantigen-specific Treg and high cell yield.

Naturally occurring CD4^pos^CD25^high^ Treg display a polyclonal TCR V-beta pattern [28]. Stimulation of this polyclonal Treg population with allogeneic stimulator APC has been shown to specifically activate alloantigen-reactive Treg in mice [1], [4], [6], [7], [29], [30] and humans [27]. Thus, these studies indicate that it is feasible to generate alloantigen-specific Treg from the naturally occurring CD4^pos^CD25^high^ Treg population. Here, we elaborated on these findings and questioned how to generate high numbers of alloantigen-specific Treg starting with polyclonal CD4^pos^CD25^high^ Treg populations obtained from peripheral blood. We specifically focused on a magnetic bead based method for Treg isolation, so as to fit in with currently available clinical grade isolation tools, with the objective to facilitate easy translation into clinical practice. Clinical grade (Good Manufacturing Practice, GMP) CD4^pos^CD25^high^ Treg isolation by a magnetic bead based method is now feasible using the CliniMACS system [31], [32], albeit that Treg purity is suboptimal (50–60%). Our recent data on expansion of CliniMACS isolated Treg confirms that the results reported here can indeed be extrapolated to clinical grade purification (unpublished observations). Clearly, with regard to purity of the starting population magnetic bead isolation is inferior to FACS sorting, but this latter method is not readily available for GMP purposes. We obtained with high purity FACS sorted Treg similar results regarding antigen specificity, but with lower expansion rates and higher overall suppressive capacity (unpublished observations).

To design a successful strategy for obtaining high numbers of alloantigen-specific Treg, we hypothesized that it might be beneficial to combine *ex vivo* expansion cycles applying polyclonal stimulation, to boost expansion, and cycles applying alloantigen stimulation, to selectively stimulate alloantigen-specific Treg. Our data show that a high degree of alloantigen-specificity could be obtained in as little as two cycles of expansion. To increase alloantigen-specificity even more, it would be an option to repeat alloantigen expansion stimulation for multiple cycles, which in theory would progressively enrich for strictly antigen-specific Treg. However, if Treg populations were expanded for more than two expansion cycles, we observed a loss of suppressive capacity and high cell death (unpublished results).

Although highest cell yields were expected after expansion with two polyclonal stimulation cycles, our data showed similar cell numbers for all strategies. This result may in part be due to the fact that we opted for a standardized 10 day culture cycle. This choice was made based on the fact that strategies comprising one or more alloantigen stimulation cycles showed optimal expansion at day 10. However, expansion of cells in a second subsequent polyclonal stimulation cycle reached an optimum at day 7, cell death occurred thereafter.

Of note, the strategies described in this report generate Treg with direct alloantigen-specificity. These cells may be especially of benefit for patients receiving an HLA mismatched stem cell graft, where this route of alloantigen-reactivity is important in graft versus host pathology [33]. Although transplantation centers pursue a high degree of HLA-matching to prevent harmful reactions, HLA-locus mismatched and haploidentical transplants are increasingly being performed [34]. In these cases current immunosuppressive regimen may benefit from the addition of an antigen-specific component. In solid organ transplantation, next to direct alloantigen recognition, the indirect route of alloantigen presentation clearly contributes to graft rejection [35]. With this in mind, it was recently shown, that it is also feasible to obtain Treg with indirect alloantigen-specificity by stimulation with autologous dendritic cells pulsed with allo-HLA-peptides [36].

Recently, the first clinical trials on Treg immunotherapy have been initiated; in these studies, either CliniMACS isolated CD4^pos^CD25^high^ Treg or *ex vivo* manipulated CD4^pos^ T cell lines containing induced regulatory Tr1 cells are being infused in patients receiving stem cell transplantations [22]. So far, no effects have been reported.

In summary, this study has shown that it is feasible to obtain human functional alloantigen-specific Treg in large numbers for immunotherapeutic purposes. This may be a valuable aid in the clinical application of Treg, aiming at clinical tolerance to selected antigens, while minimizing general immune suppression.

## Materials and Methods

### Treg expansion strategies

To determine the optimal expansion strategy for obtaining high numbers of functionally active human alloantigen-specific Treg from CD4^pos^CD25^high^ Treg, we devised four different strategies, consisting of two subsequent cycles of expansion with alternated polyclonal and alloantigen-specific stimulation (Figure 1).

### Cell preparation / isolation

Buffy coats were purchased from Sanquin bloodbank, Nijmegen, The Netherlands. These buffy coats were obtained from healthy human donors upon written informed consent with regard to scientific use.

The current study did not require approval from an ethical committee according to the Dutch Medical Research Involving Human Subjects Act (WMO). PBMC were isolated by density gradient centrifugation (Lymphoprep, Nycomed Pharma, Roskilde, Denmark). CD4^pos^ T cells were negatively selected using mAbs directed against CD8 (RPA-T8), CD14 (M5E2), CD16 (3G8), CD19 (4G7), CD33 (P67.6), CD235a (GA-R2(HIR2) (all from BD Biosciences, San Jose, CA), and CD56 (MOC-1) (Dako, Glostrup, Denmark) combined with sheep-anti-mouse-IgG coated magnetic beads (Dynal Biotech, Oslo, Norway), routinely resulting in a >90% pure CD4^pos^ T cell fraction. CD25^high^ Treg and CD25^neg^ conventional T cells were separated by MACS-sorting, using 10 μl anti-CD25 magnetic microbeads / 10^7^ CD4^pos^ cells (Miltenyi Biotec, Bergisch Gladbach, Germany). HLA typing was performed by serological and DNA based techniques according to international (ASHI/EFI) standards [37].

### CD4^pos^CD25^high^ Treg expansion

Cell cultures were performed in 96-well round bottom plates with culture medium consisting of RPMI 1640 supplemented with pyruvate (0.02 mM), penicillin (100 U/ml), streptomycin (100 μg/ml), and 10% human pooled serum (HPS), in a 37^o^C, 95% humidity, 5% CO_2_ incubator.

To optimize Treg expansion conditions, the mode and strength of stimulation, exogenous cytokine concentration, and expansion cycle length were varied in early experiments, as reported in the Results section. For assessment of cell division using CFSE, 1–5×10^6^ cells were labeled with 0.5 μM CFDA-SE (Molecular Probes) prior to stimulation. The final expansion protocols are described below.

For primary and secondary cycle alloantigen-specific expansion, 2.5×10^4^ T cells were cultured with 10^5^ irradiated (30 Gy) HLA mismatched allogeneic PBMC (target alloantigen, ratio alloPBMC∶Treg  =  4∶1).

For first cycle polyclonal expansion, 2.5×10^4^ T cells were cultured with 1.25×10^4^ anti-CD3+ anti-CD28 coated microbeads (Dynal Biotech). For secondary cycle polyclonal expansion, 2.5×10^4^ Treg were cultured with 5 μg/ml platebound anti-CD3 (UCHT1, BD Biosciences, 4 hours in PBS in incubator) plus 1 μg/ml soluble anti-CD28 (CD28.2, BD Biosciences).

Exogenous rhIL-2 (25 U/ml, Chiron, Amsterdam, the Netherlands) and rhIL-15 (10 ng/ml BioSource International, Camarillo, CA) were added to all expansion cultures. Wells were split and fresh medium containing cytokines was added every 3 days. After 10 days, the cells were harvested, washed and rested for 2 days in 5% HPS culture medium with 5 ng/ml rhIL-15 before analysis or further expansion. In all experiments, Tconv were included as control.

### Flow cytometry

The phenotype of cells was analyzed by five-color flow cytometry (FC500, Beckman Coulter, Fullerton, CA). For cell surface staining, the following conjugated mAbs were used: CD25(M-A251)-PE, CD70(Ki-24)-PE, CD127(hIL-7R-M21)-AlexaFluor647 (BD Biosciences), CD27(M-T271)-FITC (Dako), CD4-(SFCI12T4D11)-PCy7 and CD62L(DREG56)-ECD (Beckman Coulter). To stain apoptotic cells, 7-amino-actinomycin-D (7AAD, Sigma-Aldrich, Zwijndrecht, the Netherlands) was added to cells prior to acquisition. For intracellular staining, Fix and Fix/Perm buffer (eBioscience, San Diego, CA) were used according to the manufacturer's instructions in combination with FoxP3(259D/C7)-AlexaFluor647 or Bcl-2(100)-PE (BD Biosciences). Isotype controls were used for gate settings. In some experiments, cell were counted by flowcytometry using Flow-Count fluorospheres (Beckman Coulter).

### Stimulation assay to analyze T cell anergy

T cell anergy was examined in (re-)stimulation assays. 2.5×10^4^ cells were stimulated with 10^5^ irradiated allogeneic stimulator PBMC in the presence or absence of IL-2 (12.5 U/ml). Classically, T cell anergy is defined as a low proliferative capacity upon stimulation with antigen only, which can be reversed by addition of exogenous IL-2. Proliferation was measured at indicated time-points by determination of [^3^H]Thymidine incorporation as described above. Tests were set up in triplicate; results are expressed as mean+SD counts per 5 minutes.

### Co-culture suppression assay

Suppressive capacity of Treg was studied in mixed lymphocyte reaction (MLR) co-culture suppression assays. Proliferation was determined by either [^3^H]Thymidine incorporation or CFSE dilution assays.

For assessment of cell division using [^3^H]Thymidine, 5×10^4^ naive autologous CD4^pos^CD25^neg^ T responder cells were stimulated with 10^5^ irradiated allogeneic stimulator PBMC (either target PBMC or HLA mismatched third party PBMC). Treg or Tconv were titrated into the cultures. Proliferation was measured at day 5. To this end, 0.5 μCi [^3^H]Thymidine (Amersham Biosciences, Piscataway, NJ) was added to each well. After 8 hours, [^3^H]Thymidine incorporation was measured using a beta-plate counter (Packard, Canberra, Australia). Tests were set up in triplicate; [^3^H]Thymidine incorporation were expressed as mean+SD counts per 5 minutes.

For assessment of cell division using CFSE dilution, naïve autologous 5–10×10^6^ CD4^pos^CD25^neg^ T responder cells were labeled with 1 μM CFDA-SE (Molecular Probes) prior to stimulation, and allogeneic stimulator PBMC were labeled with 2 μM PKH26 (Sigma-Aldrich). 2.5×10^4^ CFSE^pos^ T responder cells were stimulated with 10^5^ PKH26^pos^ irradiated allogeneic stimulator PBMC (either target PBMC or HLA mismatched third party PBMC). Unlabeled Treg or Tconv were titrated into the cultures. At day 5, 7-amino-actinomycin-D (7AAD, Sigma-Aldrich) was added to the samples. Tresp division was analyzed by flow cytometry (FC500, Beckman Coulter, Fullerton, CA), excluding 7AAD^pos^ cells, PKH26^pos^ stimulator cells and CFSE^neg^ Treg or Tconv and expressed as percentage dividing cells.
